# Identification of Genomic Regions Contributing to Protein Accumulation in Wheat under Well-Watered and Water Deficit Growth Conditions

**DOI:** 10.3390/plants7030056

**Published:** 2018-07-11

**Authors:** Ibrahim S. Elbasyoni, Sabah M. Morsy, Raghuprakash K. Ramamurthy, Atef M. Nassar

**Affiliations:** 1 Crop Science Department, Faculty of Agriculture, Damanhour University, Damanhour 22516, Egypt; Sabahinunl@gmail.com; 2 Department of Agronomy and Horticulture, University of Nebraska-Lincoln, Lincoln, NE 68583, USA; kasturiraghuprakash@gmail.com; 3 Plant Protection Department, Faculty of Agriculture, Damanhour University, Damanhour 22516, Egypt; atef.nassar@dmu.edu.eg

**Keywords:** wheat, grain protein content, water deficit, genome-wide association mapping

## Abstract

Sustaining wheat production under low-input conditions through development and identifying genotypes with enhanced nutritional quality are two current concerns of wheat breeders. Wheat grain total protein content, to no small extent, determines the economic and nutritive value of wheat. Therefore, the objectives of this study are to identify accessions with high and low grain protein content (GPC) under well-watered and water-deficit growth conditions and to locate genomic regions that contribute to GPC accumulation. Spring wheat grains obtained from 2111 accessions that were grown under well-watered and water-deficit conditions were assessed for GPC using near-infrared spectroscopy (NIR). Results indicated significant influences of moisture, genotype, and genotype × environment interaction on the GPC accumulation. Furthermore, genotypes exhibited a wide range of variation for GPC, indicating the presence of high levels of genetic variability among the studied accessions. Around 366 (166 with high GPC and 200 with low GPC) wheat genotypes performed relatively the same across environments, which implies that GPC accumulation in these genotypes was less responsive to water deficit. Genome-wide association mapping results indicated that seven single nucleotide polymorphism (SNPs) were linked with GPC under well-watered growth conditions, while another six SNPs were linked with GPC under water-deficit conditions only. Moreover, 10 SNPs were linked with GPC under both well-watered and water-deficit conditions. These results emphasize the importance of using diverse, worldwide germplasm to dissect the genetic architecture of GPC in wheat and identify accessions that might be potential parents for high GPC in wheat breeding programs.

## 1. Introduction

Wheat (*Triticum aestivum* L.) is the food commodity for more than third of the world's population. Wheat grain is a rich source of starch (carbohydrate). Therefore wheat is primarily considered as a source of energy [1]. However, wheat grain contains also moderate amounts of dietary proteins which determines, to a large extent, both the end-use quality and wheat grain price [2]. Wheat grain total protein content (GPC) ranges from 9 to 15% of the dry weight [3,4]. Although, GPC depends primarily on the genotype; the environment and genotype × environment interaction also plays an essential role in grain protein accumulation [5]. 

Nitrogen fertilization is the critical environmental factor that affects protein accumulation; if nitrogen fertilization stays constant, increased yield often results in decreased protein content because of nitrogen dilution by the large biomass [6,7,8,9]. Furthermore, under stress conditions, grain protein content tends to be higher compared to either irrigated or nitrogen-limited conditions [10]. Water deficit increases grain total protein content, but it decreases grain yield [11]. Wheat genotypes with higher yield potential tend to have lower protein content and *vice versa* [12]. Several explanations for the negative relationship between grain protein content and yield have been proposed [13]. However, some wheat genotypes deviate from the previous relationship, i.e., they produce high yield and high grain protein content [14]. That deviation implies that the nitrogen supply to grains was increased, but it was not associated with a reduction in the grain yield [15]. 

Exploring genetic resources to identify wheat genotypes with high grain protein content is the most efficient way to improve the nutritional value of wheat grains [16]. Wheat breeders were successful in selecting genotypes with a high total protein content that were generated from cultivated materials such as “Atlas,” “Atlas66” and “Nap Hall” [17]. Previous studies reported higher GPC in landraces and wild relatives compared to modern wheat genotypes [18,19]. A wild emmer (*Triticum turgidum var dicoccoides*) genotype was identified in Israel, i.e., “FA15-3” which was found to be able to accumulate 40% protein when given adequate nitrogen fertilization [20]. High grain protein content gene *GPC-B1* allele which was originally identified in wild emmer wheat, was transferred to a spring wheat genotype and increased grain total protein content by 3% [21]. The *GPC-B1* allele accelerates senescence and increases mobilization of nitrogen, zinc, and iron to the developing grains [22]. Thus, accessions containing this allele most likely will contain high protein as well as high iron and zinc [23]. However, most of the modern tetraploid and hexaploid wheat genotypes have lost a functional allele of *GPC-B1* [16]. During the last decade, several QTLs for GPC were mapped using association mapping (AM) and biparental populations on chromosomes 5A, 5D, 2D, 2B, 6A, 6B and 7A [24,25,26,27,28,29,30,31], that QTLs were validated and used in marker-assisted selection to improve GPC.

Marker-assisted selection (MAS) was defined as one of the promising avenues to improve wheat total protein content and grain yield [32]. The critical step in MAS is to identify molecular markers associated with desirable phenotypic traits using AM or biparental populations [33]. Association mapping (AM) can be applied to structured populations [34], thus incorporating a broad spectrum of germplasm is possible [34,35,36]. However, the successful application of association mapping requires comprehensive phenotypic and genotypic data. The dramatic decrease in the genotyping costs [37] in addition to the availability of high throughput phenotyping technologies such as Near-Infrared Spectroscopy (NIR) make AM a viable approach for large populations [38]. Furthermore, A robust sequence and annotation of the wheat genome are now available [39] with the latest developments in genomic technologies. This might allow researchers to identify new loci associated with GPC genes and dissect the genetic architecture of GPC in wheat.

Three strategies were adopted to select for high GPC and grain yield, i.e., selecting for high grain protein alone, selecting for high grain protein within highest yielding genotypes, and using an index to simultaneously select for both protein and yield [40]. In the current study, the most recent developments in genotyping and phenotyping technologies were applied to identify genomic regions associated with GPC and select accessions with high and low grain protein content using a worldwide collection of spring wheat accessions. 

## 2. Materials and Methods

### 2.1. Plant Materials and Field Growth Conditions

Wheat grains obtained from 2111 wheat accessions (882 landraces; 493 breeding lines; 419 cultivars and 317 with uncertain category) were used in the current study. The accessions seeds were provided by the national small grains collection (NSGC) located in Aberdeen, ID, USA. The accessions were screened in Egypt during 2015/2016 and 2016/2017 growing seasons for total protein content under well-watered and water deficit conditions in Damanhour university experimental farm (30°45′19.4″ N, 30°29′4.8″ E). During the two growing seasons, drought stress was imposed by controlling irrigation during the reproductive stage in which plants were irrigated at 40% depletion of plant available water (PAW) (well-watered), or 80% PAW (water deficit). Well-watered and water deficit treatments were applied on two sublocations within the same experimental farm to facilitate the control of water application. For both sublocations, the wheat accessions were planted in two replicates using a randomized incomplete block design [41] in plots of four rows wide with 25 cm between rows and two meters long. The incomplete blocks consisted of 50 accessions in addition to three check cultivars, i.e., “Sids13”, Gimmiza 9”, and “Giza 168. The check cultivars were planted in each incomplete block. 

### 2.2. Estimation of Grain Protein Content (GPC)

Grain protein content (% or g/100 g) was estimated using near-infrared spectroscopy (NIR) with a Perten DA7250 diode array NIR (Springfield, IL, UAS). NIR is a nondestructive technique that complies with the ISO 12099 standard method. The measurements of GPC were done in the near infrared region 950–1650 nm and readings were processed in NetPlus software (Perten, Hägersten, Sweden), which includes validation calculation modules, such as calculations of bias, slope, and standard errors of prediction against the reference methods. However, for initial calibration of the Perten DA7250, the crude protein content of 100 wheat accessions was measured using the Kjeldahl method (Pelican Equipment’s, Chennai, India). The correlation coefficient (r) between the calibration set and Perten DA7250 NIR readings was 0.964 for crude protein (% dry basis).

### 2.3. Single Nucleotide Polymorphism (SNP)

Wheat accessions included in this study were genotyped through the Triticeae Coordinated Agriculture Project (TCAP) using the Illumina iSelect 9 K (Illumina, Madison, WI, USA) wheat array [42] at the USDA-ARS genotyping laboratory in Fargo, ND, USA. The single nucleotide polymorphism (SNP) markers were filtered by removing SNPs with missing values >10% and minor allele frequency (MAF) <5%. The filtration step resulted in 5090 high-quality SNPs in which the missing values were imputed using random forest regression [43], which was applied using the MissForest R/package [44]. Then, the filtered and imputed SNP markers were used for the association mapping analysis, in which SNP markers were plotted in a Manhattan plot using “WNSP 2013 consensus map”; available on: (https://triticeaetoolbox.org/wheat/) according to Wang et al. [45].

### 2.4. Statistical Analysis

Analysis of variance was carried out by fitting the following model [46]:

*Y_ijlm_* = *µ* + *E_i_* + *EB*_(*il*)*j*_ + *G_m_* + *EG_im_* + *ԑ_ijlm_*

where *Y_ijlm_* is the response measured on the *_ijlm_* plot, *µ* is the overall mean, *E_i_* is the effect of *i*th environment, *EB*_(*il*)*j*_ is *j*th incomplete block nested within *l*th complete block and *i*th environment (random), *G_m_* is the effect of *m*th accession, *EG_im_* is the interaction effect among *i*th environment and *m*th accession, and *ԑ_ijlm_* is the experimental error. Type III expected mean square estimation was conducted as follows:

SourceType III Expected Mean SquareEnvironment (Env)Var (Error) + 45.372 Var (IBlock (Env × Rep)) + Q (Env, Env × Genotypes)Incomplete block (Env × block)Var (Error) + 36.829 Var (IBlock (Env × Rep))AccessionsVar (Error) + Q (Genotypes, Env × Genotypes)Env × AccessionsVar (Error) + Q (Env × Genotypes)

Homogeneity and normality of variance were checked using Bartlett and Shapiro-Wilk statistics using R/package agricolae [47]; Least Square Means (Lsmeans) were estimated using R/package lsmeans [48]. Lsmeans were compared using Tukey's studentized range (HSD) (at *p*-value < 0.05). Pearson correlation analysis (*r*) was carried out between lsmeans using R/package corr.test [47]. Mean-based heritability (h^2^) was estimated using the following model:

h^2^ = σ_G_^2^/[σ_G_^2^ + (σ_E_^2^/*ri*)]

where σ_G_^2^ is the genetic variance, σ_E_^2^ the residual variance and *ri* is the number of replicates [49].

### 2.5. Association Mapping

The estimated Lsmeans for GPC and SNP markers were subjected to association analysis according to the following mixed linear model (MLM) in R package GAPIT [50].

*Y* = *μ* + *Zu* + *Wm* + *e* (1)

where *Y* is a vector of the total protein content, *μ* is a vector of intercepts, *u* is an n × 1 vector of random polygene background effects, *e* is a vector of random experimental errors with mean 0 and covariance matrix Var(*e*), *Z* is an incidence matrix relating *Y* to *u*. Var(*u*) = 2 KVg, where K is a known n × n matrix of a realized relationship matrix, estimated using the A.mat function in R software [51], as K = WW/C, where *W*_ik_ = X_ik_ + 1 − 2_pk_ and _pk_ is the frequency of the one allele at marker _k_ [51], Vg is the unknown genetic variance, which is a scalar, *m* is a vector of fixed effects due to SNP markers, *W* is incidence matrix relating *Y* to *m*. Var(*e*) = RVR, where R is an n × n matrix, and VR is the unknown residual variance, which is a scalar too. Furthermore, principal component analysis (PCA) was conducted using the filtered SNP markers [52] and the integrated PCA function (prcomp) of the R software. In addition to Model (1), another three models were fitted. Model (2) contained the K matrix and the first PCA; Model (3) contained the K matrix, in addition to PCA1 and 2. Moreover, Model (4) contained the K matrix, in addition to the first three PCAs. *p*-values estimated from the mixed models were subjected to false discovery rate (FDR) corrections using Q-value estimates applied in the R package *q*-value [53]. The proportion of phenotypic variance explained (R^2^) by the significant markers, and their additive effects were estimated using the GAPIT function, according to Wray et al. [54], in R software [50].

## 3. Results

### 3.1. Grain Protein Content (GPC)

**Figure 1:**
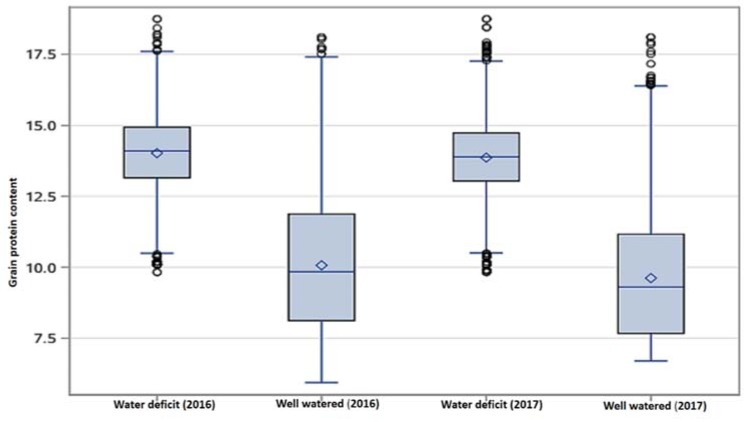
Boxplot for the overall performance of the 2111 wheat accessions across the four environments (well-watered and water-deficit conditions in 2016 and 2017 growing seasons).

Normal distribution and homogeneity of variance for grain protein content (GPC) were observed across the four environments (two seasons and two water regimes). Thus, combined analysis of variance across environments was conducted. Combined analysis of variance for GPC indicated a highly significant effect (*p*-value < 0.01) for the environments, genotypes, and genotype × environment interaction (Table 1). Broad-sense heritability estimates ranged from 0.49 to 0.60 for well-watered and water deficit conditions, respectively. Furthermore, the broad sense heritability estimates across years, and water regimes (the four environments) was 0.64 lsmeans of the grain protein content (GPC) ranged from 5.96 to 17.11% with a mean of 10.15 under well-watered conditions during 2016, and 6.88 to 17.43 with a mean of 9.67 in 2017 growing seasons. On the other hand, under water deficit conditions, GPC ranged from 11.12 to 18.5 with a mean of 14.9 in 2016 and 9.8 to 18.3 with a mean of 13.97 in 2017 growing seasons. Although, no significant difference was detected for the difference between means of the growing seasons, the difference between the lsmeans of the water regimes was highly significant, based on HSD at 0.01 probability level. Overall, our results indicated that water deficit increased GPC by 29% across the two growing seasons (Figure 1).

**Figure 2:**
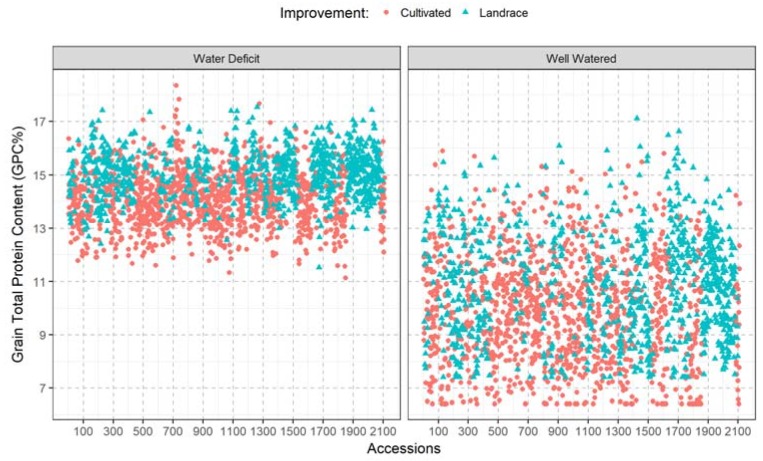
The overall performance of the 2111 wheat accessions across the two growing seasons under water deficit and well-watered growth conditions.

Furthermore, the correlation between GPC obtained from well-watered with that obtained water deficit across all genotypes was positive and significant (*r* = 0.23, *p*-value = 0.01). The first quartiles for the GPC across growing seasons (the cut off for the lowest 25%) under well-watered and water deficit conditions were ≤8.36 and 13.41, respectively (Figure 1). Whereas, the third quartile (the cut off for the highest 25%) of the genotypes under well-watered and water deficit conditions were ≥11.35 and 14.66, respectively. The first and third quartiles in this study were used as criteria to classify the genotypes into high and low GPC genotypes. Therefore genotypes with GPC ≤8.36 under well-watered and ≤13.41 under water deficit conditions, were defined as low protein genotypes. On the other hand, genotypes with GPC ≥11.35 under well-watered and ≥14.66 under water deficit conditions were defined as high protein genotypes. Grain protein content (GPC) for all genotypes under well-watered and water deficit conditions (Figure 2) indicated that 166 (7.8% of the genotypes) had high protein content under well-watered and water deficit conditions concurrently. Another, 200 genotypes (9.47%) were classified as low protein genotypes under both well-watered and water deficit conditions concurrently. The top 20 accessions with the highest GPC under well-watered and water deficit conditions are presented in Table 2, in which no overlapping accessions between the two water regimes were detected. Out of the top 20 accessions, detected under well-watered conditions, nine landraces were present. On the other hand, 18 landraces were present among the top 20 accessions detected under water deficit conditions. Overall, the estimated lsmeans from the landraces (882 accessions) under well-watered conditions was 10.9; which was 11.22% higher than the overall average of all other accessions (Table 2). Additionally, under water deficit conditions the average GPC for the landraces was 15.04 which was 7.9% higher than the overall average of all other accessions. Overall, our results indicate that moisture has a significant impact on GPC accumulation in wheat. Landraces had higher GPC, compared to other germplasm used in the current study.

### 3.2. Association Mapping for Grain Protein Content

A total of 3215 mapped SNPs were used for estimating the extent of linkage disequilibrium (LD) in the 2111 wheat accessions. Only SNP loci having MAF ≥0.05 and missing values ≤10% were used to estimate r^2^ across all SNPs. The estimates of r^2^ for all pairs of SNPs loci were used to determine the rate of LD decay with genetic distance. Across the three wheat genomes, i.e., A, B and D using only markers with significant r^2^ (*p*-value = 0.001), the LD ranged from 0 to 0.35. Overall, LD declined to 50% of its initial value at about 8 cM (Supplementary Materials, Figure S1). Eigenvector decomposition of the kinship matrix was used to investigate the population structure among accessions. The first principal component (PCA) accounts for less than 1% of the total variance (Supplementary Materials, Figure S2). Nevertheless, GWAS models with kinship matrix (K matrix, supporting information Figure S3) with zero, one, two or three PCAs were compared using Bayesian information criteria (BIC). The results indicated noticeable difference between the four models. Additionally, the first model, i.e., with no PCA produced the highest BIC values, given that the largest is the best [55]. Therefore, we reported the results of association mapping using only the K matrix in which it accounted for most of the stratification among accessions. 

**Figure 3:**
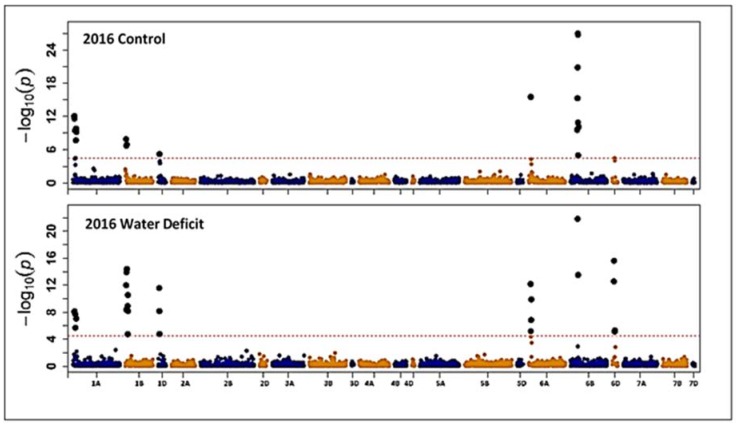
Manhattan plot for grain protein content (GPC) obtained from genome-wide association mapping in the 2016 growing season.

**Figure 4:**
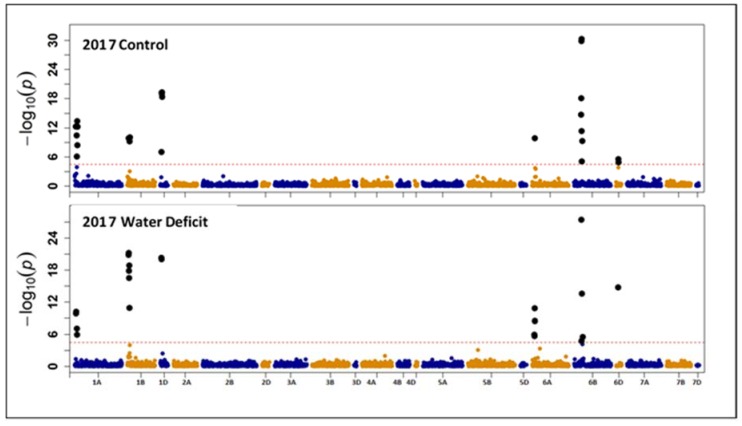
Manhattan plot for grain protein content (GPC) obtained from genome-wide association mapping in the 2017 growing season.

Association mapping analysis was conducted on each environment separately (two growing seasons and two water regimes). Genome-wide association mapping (GWAS) indicated that 46 SNP markers found to be significantly linked with GPC. The significant SNP markers were located on chromosomes 1A (12 SNPs), 1B (12 SNPs), 1D (7 SNPs), 6A (6 SNPs), 6B (7 SNPs) and 6D (3 SNPs) (Figure 3 and Figure 4). Out of the 46 significant SNP markers, ten markers were linked with GPC under well-watered and water deficit conditions in one growing season at least. Three SNP markers (IWA3169, IWA3501, and IWA7937) were significantly linked with GPC across the four environments (2016, 2017 growing seasons, and well-watered and water deficit conditions) (Table 3). Four markers (IWA6649, IWA6787, IWA3481 and IWA4351) found to be linked with GPC in three environments (2016 well-watered, 2016 and 2017 water deficit conditions) (Table 3). These results together indicate that some loci were significantly associated with GPC in wheat irrespective of water status.

Under well-watered conditions for the two growing seasons, seven SNP markers (IWA5150, IWA4643, IWA4754, IWA3923, IWA6466, IWA6467, and IWA5986) found to be significantly linked with GPC. On the other hand, under water deficit conditions for the two growing seasons, six SNP markers (IWA7191, IWA8199, IWA7345, IWA3446, IWA7288, and IWA7287) found to be significantly linked with GPC. In contrary, 14 markers (IWA4753, IWA4678, IWA4644, IWA4506, IWA4163, IWA3738, IWA5020, IWA5019, IWA5018, IWA4598, IWA4551, IWA4552, IWA4962, and IWA4730) found to be significantly linked with GPC during only one growing season under well-watered conditions. Another ten markers (IWA7616, IWA3624, IWA6673, IWA7007, IWA8551, IWA6610, IWA6611, IWA7480, IWA7048, and IWA7050) found to be significantly linked with GPC under water deficit conditions in only one growing season. Repeatability of the GPC associated loci in 2 seasons under any given water treatment suggests the feasibility of using/developing markers in LD with these loci.

## 4. Discussion

Protein content is an essential compositional trait in wheat, which has a broad impact in the food industry concerning human nutrition and health. Consequently, breeding for enhanced end-use quality is one of the essential breeding goals in wheat. However, GPC in wheat is positively affected by water deficit compared to well-watered conditions [10]. In this study, we seek to evaluate a comprehensive spring wheat collection for grain protein content (GPC) and to locate genomic regions associated with GPC under well-watered and water deficit conditions using GWAS approach. 

The most striking observation in this study was the weak, positive and significant correlation between GPC obtained from the well-watered condition and water deficit conditions (*r* = 0.23). That weak correlation implies strong genotype × environment interaction, in which genotypes responded differently concerning water treatment. Increase in GPC under water deficit conditions could be mainly due to higher rates of accumulation of grain nitrogen and lower rates of accumulation of carbohydrates. High moisture, on the other hand, may decrease GPC by dilution of nitrogen with carbohydrates [56]. An increased grain protein and gluten content in response to water deficit as compared to the well-watered experiment in a winter wheat was also reported in a previous study [57]. The current study, as well as previous reports, indicated a significant effect of environment (moisture and growing seasons) on wheat GPC accumulation. Analysis of variance indicated a significant effect of moisture, genotype, and genotype × environment interaction on GPC in wheat, suggesting that GPC is a complex trait influenced by several factors. The significant genotypic effect observed in this study also indicated a wide range of variation for GPC accumulation among wheat accessions used. Moreover, around 366 (166 with high GPC and 200 with low GPC) wheat genotypes performed relatively the same across environments, which implies that GPC accumulation on these genotypes was less responsive to moisture.

Genotypic variation is a result of several alleles on genes which result in different responses to environmental conditions [58]. Furthermore, landraces serve as a valuable genetic resource in which it might provide new alleles for improvement of economically important traits such as GPC [19]. Results reported herein showed that landraces outperformed cultivated genotypes concerning GPC. These findings agree with previous reports [59,60] in which 121 landraces, 101 obsolete cultivars, and modern wheat cultivars were evaluated for GPC under the same environmental conditions, and landraces had higher total protein content compared to other studied accessions. Grain quality of some wheat landraces should be of particular interest because much broader diversity can be found in landraces compared to modern wheat cultivars [61]. Additionally, most of the organic wheat production systems rely on cultivars that were developed for high-input production systems [60,62]. Wheat landraces have been developed mostly in environments with low nutrient availability; they represent a source of variation for selection of genotypes adapted to cropping systems with low fertilizer input [61]. In the current study, we identified 224, 214 and 70 wheat landraces that were found to have high GPC under well-watered, water deficit and both conditions, respectively. Our results and previous reports indicated that GPC depends mainly on genotype, environment, and genotype × environment interaction [59]. However, the response mechanism that modifies protein accumulation under water deficit conditions is still unclear. Recently, a putative mechanism underlying the increased accumulation of storage proteins in wheat endosperm under water deficit was provided by Chen et al. [63]. They identified four differentially expressed miRNAs induced by drought stress that may affect the development of protein bodies in caryopsis by regulating the expression levels of target genes involved in protein biosynthesis pathways.

One of the primary goals of this study was to locate significant genomic regions that control the accumulation of GPC which might shed light on the genetic architecture of GPC and the protein accumulation mechanism. The genome-wide association mapping analysis, applied in the current study, using the kinship (K) matrix in a mixed model indicated that K matrix was adequate in accounting for population structure [64]. Also, these results agree with those of Zhao et al. [65], in which they found that K models were adequate for genome-wide association mapping. Furthermore, the K model was more effective in reducing the false-positive rate compared to using the Q + K model. Linkage disequilibrium (LD) was estimated using r^2^ among all pairs of SNPs loci, in which r^2^ in this study was 0.09, which is higher than that obtained by Breseghello and Sorrells [66] and 0.019 reported by Neumann et al. [67] because of their small size populations, and with a similar number of marker pairs. This indicates that the population size might have an impact on the LD. 

Genome-wide association analysis (GWAS) was conducted on each environment separately to measure the repeatability of the significant SNPs, and the effect of moisture on the genomic regions controlling GPC. Several SNPs found to be significantly linked to the GPC under well-watered conditions but not significantly linked to GPC under water deficit conditions and *vice versa*. Moreover, ten QTLs were linked with GPC under both well-watered and water deficit conditions. The GWAS analysis suggested a significant role of genotype × environment interaction in detecting GPC associated loci. Genome-wide association studies using diverse wheat germplasm have successfully detected GPC associated loci in durum wheat [68], and bread wheat lines [69]. Thus, the SNPs associated with GPC under water deficit or well-watered environmental conditions, from this study might provide useful molecular information for wheat breeders to incorporate specific QTLs to increase GPC in low input or drought-stressed environments. Around 50% of the significant SNPs detected in the current study was on chromosome 1, where copies of *Glu-B1* and *Gli-B1* genes reside [70]. *Glu-B1* and *Gli-B1* genes were previously reported to contribute of about 24.6 and 19.5% of the total phenotypic variation for sedimentation volume (determines gluten strength and in turn cooking quality of pasta) [2]. Several SNP loci in LD with sedimentation volume were discovered recently on chromosome 1A and 1B, in durum wheat [68]. 

These results together emphasized the importance of using diverse worldwide germplasm to dissect the genetic architecture of GPC in wheat and identify accessions that might be potential parents in wheat breeding programs. Ongoing multiple years, multiple replication study using 406 accessions identified in the current study is being conducted, to evaluate these genotypes for yield and validate the GPC associated loci detected herein. Furthermore, GPC estimates under well-watered and water deficit conditions was used as a selection parameter to downsize the number of accessions from 2111 to 406. Reducing the number of accessions will allow us to profoundly investigate other wheat quality aspects such as concentrations (soluble and insoluble) of glutenin, α/β, γ gliadin and albumin/globulin in addition to the total protein for high and low GPC genotypes.

## 5. Conclusions

Based on previous research and our findings, the spring wheat collection used in this study contains high protein accessions. Furthermore, GPC measurement under well-watered and water deficit conditions was used as a selection criterion to reduce the number of accessions from 2111 to 406 accessions. This reduction in the number of studied accessions will allow us to profoundly study other wheat quality aspects such as concentrations (soluble and insoluble) of glutenin, α/β, γ gliadin and albumin/globulin in addition to the total protein for high and low GPC genotypes. It also represents a precious resource for further investigations including annotation of relevant genomic regions/genes using available wheat genomic resources to study the GPC. Results of GWAS indicated that several genomic regions were involved in GPC accumulation in wheat grains. Furthermore, GWAS results also suggested a significant role for genotype x environment interaction in the identification of GPC associated loci under well-watered and water deficit conditions. The identified loci might allow development of marker-assisted selection (MAS) for GPC and might also facilitate the development of a better understanding of the genetic architecture that controls GPC in wheat. Therefore, the high and low GPC accessions identified in the current study were included in ongoing multiple years and locations studies to evaluate them for yield and confirm the GPC associated loci detected.

## Figures and Tables

**Table 1:** Analysis of variance for grain protein content (GPC) of the 2111 genotypes across environments.

Source	DF	Type III SS	Mean Square	F Value
**Environment**	3	70,093.78	23,364.59	19,188.5 **
**IBlock (Replicate Environment)**	256	1361.56	5.31	4.37
**Genotypes**	2113	26,096.19	12.35	10.14 **
**Environment × Genotypes**	6255	26,164.38	4.18	3.44 **
**Error**	9208	11,211.99	1.21	

**: Significant at 0.01 probability level.

**Table 2:** Lsmean values of the grain protein content (GPC) of 20 accessions with the highest values across 2015/2016 and 2016/2017 growing seasons obtained from well-watered (control) and water-deficit conditions.

Well-Watered				Water Deficit			
Accession	Origin	Improvement	Mean	Accession	Origin	Improvement	Mean
**534,406**	Algeria	landrace	14.78	**366,801**	Afghanistan	landrace	17.965
**534,448**	Algeria	landrace	15.63	**350,850**	Austria	landrace	18.38
**338,364**	Belgium	cultivar	14.83	**350,820**	Austria	landrace	18.1125
**481,731**	Bhutan	landrace	14.69	**565,254**	Bolivia	landrace	17.9275
**14,261**	Canada	breeding	15.39	**374,243**	Chad	landrace	18.135
**313,109**	Colombia	uncertain	15.13	**57,825**	India	landrace	18.0175
**372,434**	Cyprus	landrace	14.90	**382,048**	Iran	landrace	18.535
**428,672**	Czech Republic	cultivar	15.33	**625,916**	Iran	landrace	18.43
**254,023**	Europe	uncertain	15.27	**623,758**	Iran	landrace	18.055
**278,279**	Greece	landrace	15.09	**624,992**	Iran	landrace	18.03
**468,988**	Greece	landrace	16.11	**624,124**	Iran	landrace	17.9125
**15,396**	Lebanon	uncertain	15.89	**626,116**	Iran	landrace	17.9075
**520,369**	Mexico	breeding	15.80	**623,968**	Iran	landrace	17.8525
**525,283**	Morocco	landrace	15.49	**70,704**	Iraq	landrace	18.42
**477,901**	Peru	landrace	15.05	**191,987**	Portugal	landrace	18.3475
**370,724**	Poland	cultivar	15.03	**345,474**	Serbia	landrace	18.3975
**155,119**	Russian Federation	cultivar	15.68	**225,424**	Uruguay	breeding	18.355
**479,700**	South Africa	cultivar	15.48	**225,519**	Uruguay	breeding	17.8375
**241,596**	Taiwan	cultivar	15.31	**36,500**	Uzbekistan	landrace	17.95
**534,366**	Tunisia	landrace	14.98	**24,485**	Uzbekistan	landrace	17.85

**Table 3:** SNP markers that found to be significantly linked with GPC under well-watered (control) and water deficit conditions.

Marker	Chrom	Position	Well-Watered		Water Deficit		R^2^ (%)	Additive Effect	MAF	Marker	Chrom	Position	Well-Watered		Water Deficit		R^2^ (%)	Additive Effect	MAF
			2016	2017	2016	2017							2016	2017	2016	2017			
**IWA5150**	1A	9.9	+	+	−	−	0.893	−0.007	0.19	**IWA8551**	1D	32.8	−	−	+	−	1.069	0.062	0.25
**IWA6649**	1A	11.6	+	−	+	+	1.141	0.07	0.35	**IWA3481**	1D	45.1	−	+	−	+	1.122	0.13	0.07
**IWA4351**	1A	11.6	+	−	+	+	1.089	0.062	0.35	**IWA3446**	1D	45.1	−	−	+	+	1.001	0.086	0.07
**IWA4643**	1A	21	+	+	−	−	0.892	0.004	0.28	**IWA5020**	1D	47.7	−	+	−	−	0.918	−0.026	0.33
**IWA4753**	1A	21.7	−	+	−	−	0.9	−0.02	0.1	**IWA5019**	1D	47.7	−	+	−	−	0.918	−0.026	0.33
**IWA7191**	1A	21.7	−	−	+	+	0.965	0.053	0.13	**IWA5018**	1D	47.7	−	+	−	−	0.917	−0.026	0.33
**IWA4678**	1A	22.5	−	+	−	−	0.901	0.028	0.08	**IWA4598**	1D	48.6	−	+	−	−	0.912	0.023	0.33
**IWA4644**	1A	22.9	−	+	−	−	0.915	−0.026	0.16	**IWA7007**	6A	10	−	−	+	−	0.918	0.025	0.23
**IWA4754**	1A	23.2	+	+	−	−	0.892	0.003	0.34	**IWA4551**	6A	16.2	+	−	−	−	1.294	−0.13	0.13
**IWA4506**	1A	26.9	+	−	−	−	0.907	−0.019	0.29	**IWA4552**	6A	16.2	+	−	−	−	1.313	−0.131	0.13
**IWA7050**	1A	32.5	−	−	+	−	1.305	0.092	0.38	**IWA7288**	6A	17.8	−	−	+	+	1.316	0.093	0.35
**IWA4163**	1A	32.8	+	−	−	−	1.17	−0.076	0.39	**IWA7287**	6A	21.9	−	−	+	+	1.391	0.116	0.21
**IWA4349**	1B	13.2	+	−	+	−	1.589	0.128	0.26	**IWA4962**	6A	22.8	−	+	−	−	0.923	−0.027	0.24
**IWA6787**	1B	13.2	+	−	+	−	1.433	0.105	0.28	**IWA4730**	6B	48.5	+	−	−	−	0.922	0.046	0.06
**IWA7048**	1B	22.9	−	−	+	−	1.746	0.244	0.07	**IWA3501**	6B	48.8	+	+	+	+	2.681	0.213	0.39
**IWA7480**	1B	22.9	−	−	−	+	1.472	0.111	0.35	**IWA7937**	6B	48.8	+	+	+	+	2.654	0.187	0.37
**IWA3169**	1B	23.7	+	+	+	+	2.027	0.161	0.31	**IWA3923**	6B	48.8	+	+	−	−	1.208	0.117	0.11
**IWA8199**	1B	27.4	−	−	+	+	1.271	0.099	0.25	**IWA6466**	6B	48.8	+	+	−	−	1.448	0.131	0.18
**IWA7345**	1B	28.1	−	−	+	+	1.808	0.23	0.09	**IWA6467**	6B	48.8	+	+	−	−	1.455	0.132	0.18
**IWA6611**	1B	28.1	−	−	−	+	1.636	0.131	0.27	**IWA5986**	6B	50.8	+	+	−	−	0.892	0.005	0.24
**IWA6610**	1B	28.1	−	−	−	+	1.642	−0.13	0.27	**IWA6673**	6D	17.2	−	−	+	−	1.147	0.079	0.24
**IWA3738**	1B	28.2	−	+	−	−	1.451	0.12	0.22	**IWA3624**	6D	17.3	−	−	+	−	1.073	0.068	0.41
**IWA8275**	1B	28.2	−	−	+	−	1.618	0.128	0.27	**IWA7616**	6D	29.8	−	−	−	+	1.542	0.142	0.17

− and + refer to nonsignificant and significant SNPs, respectively.

## Supplementary Materials

The following are available online at http://www.mdpi.com/2223-7747/7/3/56/s1, Figure S1: Decay of r^2^ as a function of genetic distance between SNP markers estimated for 2111 spring wheat collection from different geographic regions, Figure S2: The percentage of variance explained by principal components (PCA), Figure S3: Heatmap and dendrogram of a kinship matrix estimated using the A.mat function (rrBLUP package) based on 5090 SNPs among 2111 wheat accessions.
