# Supplementary material for: Identification of Genomic Regions Contributing to Protein Accumulation in Wheat under Well-Watered and Water Deficit Growth Conditions
Source: Plants (Basel). 2018 Jul 11;7(3):56. doi: 10.3390/plants7030056 (PMC6160930; doi:10.3390/plants7030056)
Supplement: Supplementary file 1 [file plants-07-00056-s001.zip › plants-319910-SI.pdf]

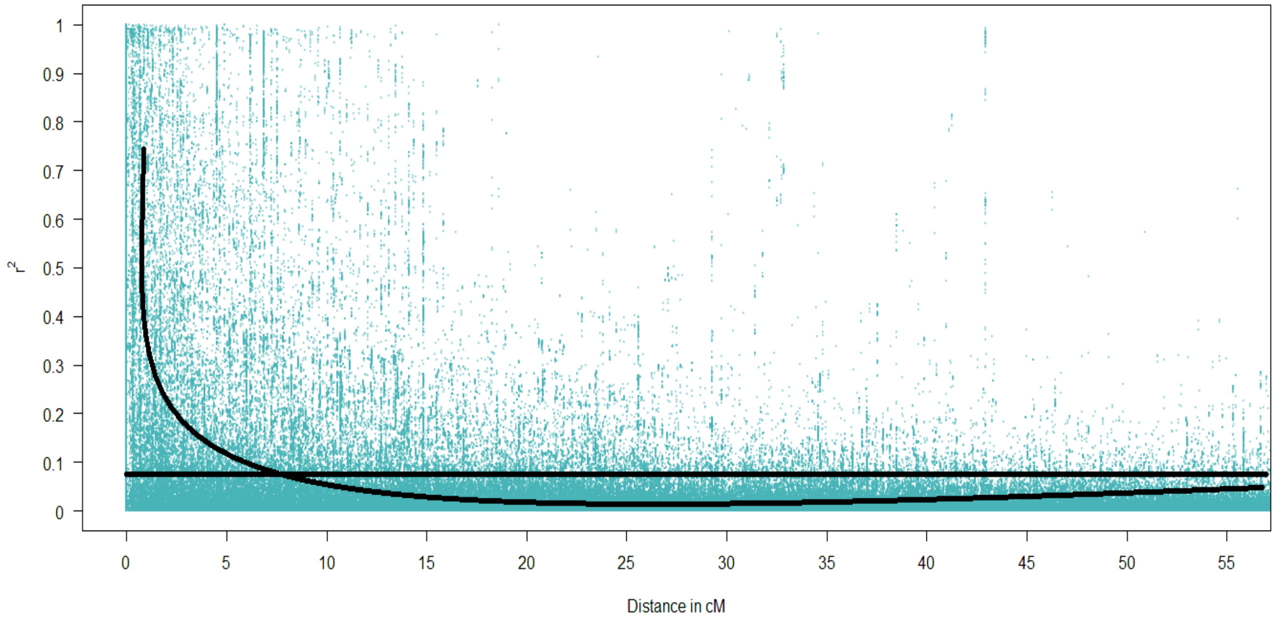

Figure S₁: Decay of r^2 as a function of genetic distance between SNP markers estimated for 2111 spring wheat collection from different geographic regions.

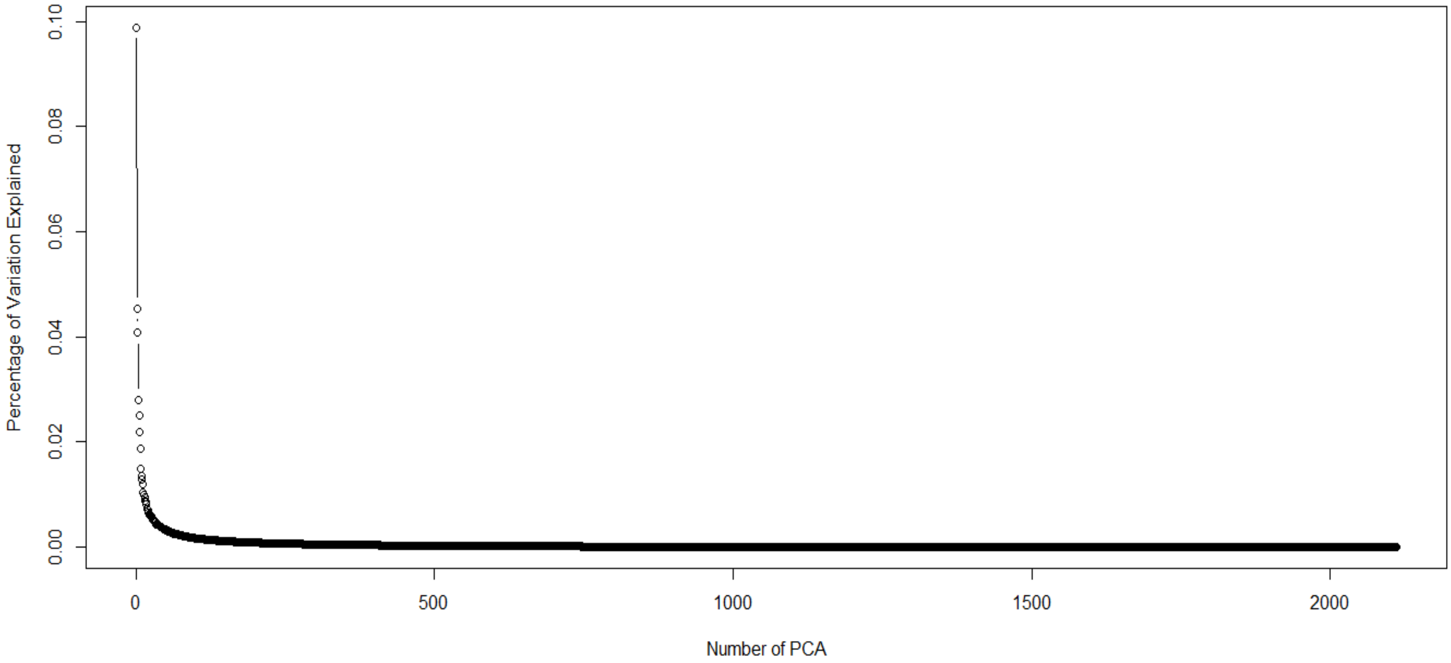

Figure S_2: The percentage of variance explained by principal components (PCA**)**

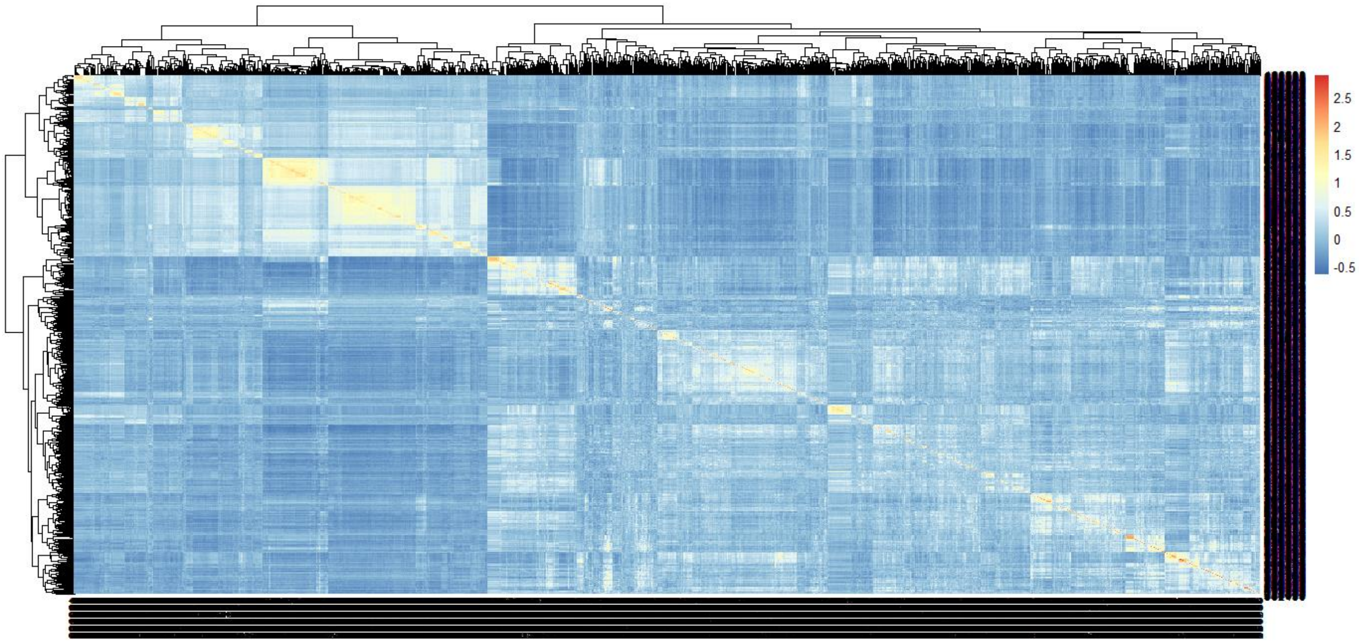

Figure S3: Heatmap and dendrogram of a kinship matrix estimated using the A.mat function (rrBLUP package) based on 5090 SNPs among 2111 wheat accessions
